# Basal Glutathionylation of Na,K-ATPase *α*-Subunit Depends on Redox Status of Cells during the Enzyme Biosynthesis

**DOI:** 10.1155/2016/9092328

**Published:** 2016-04-27

**Authors:** Vladimir A. Mitkevich, Irina Yu. Petrushanko, Yuri M. Poluektov, Ksenia M. Burnysheva, Valentina A. Lakunina, Anastasia A. Anashkina, Alexander A. Makarov

**Affiliations:** Engelhardt Institute of Molecular Biology, Russian Academy of Sciences, Vavilov Street 32, Moscow 119991, Russia

## Abstract

Many viruses induce oxidative stress and cause S-glutathionylation of Cys residues of the host and viral proteins. Changes in cell functioning during viral infection may be associated with glutathionylation of a number of key proteins including Na,K-ATPase which creates a gradient of sodium and potassium ions. It was found that Na,K-ATPase *α*-subunit has a basal glutathionylation which is not abrogated by reducing agent. We have shown that acute hypoxia leads to increase of total glutathionylation level of Na,K-ATPase *α*-subunit; however, basal glutathionylation of *α*-subunit increases under prolonged hypoxia only. The role of basal glutathionylation in Na,K-ATPase function remains unclear. Understanding significance of basal glutathionylation is complicated by the fact that there are no X-ray structures of Na,K-ATPase with the identified glutathione molecules. We have analyzed all X-ray structures of the Na,K-ATPase *α*-subunit from pig kidney and found that there are a number of isolated cavities with unresolved electron density close to the relevant cysteine residues. Analysis of the structures showed that this unresolved density in the structure can be occupied by glutathione associated with cysteine residues. Here, we discuss the role of basal glutathionylation of Na,K-ATPase *α*-subunit and provide evidence supporting the view that this modification is cotranslational.

## 1. Introduction

Viral infections lead to disruption of the redox status of mammalian cells. As a rule, the infections cause oxidative stress [1] and change the thiol redox status, which depends on the ratio of reduced (GSH) and oxidized (GSSG) glutathione [2, 3]. Under normal conditions, the reduced glutathione level in cells (1–5 mM) is 100-fold higher than the oxidized glutathione level. Under oxidative stress, this ratio can be reduced to 1 [4]. The shift of intracellular redox conditions to the oxidized state induces protein glutathionylation that protects the thiol groups of proteins from irreversible oxidation and changes their function [5, 6]. Many viruses that cause oxidative stress induce glutathionylation not only in host proteins but also in their own viral proteins. For example, the HIV-1-protease critical for viral maturation is activated by glutathionylation [7, 8]. Changes in functioning of cells during viral infection may be associated with glutathionylation of a number of key proteins [5, 6]. Notably, the acute (Flu) and chronic (hepatitis) viral infections can lead to the activation of factor Nrf/ARE [9, 10], which causes activation of glutathione transferase participating in glutathionylation of proteins [11, 12].

Na,K-ATPase creates a gradient of sodium and potassium ions necessary for all living mammalian cells. This protein is sensitive to changes in the redox status of cells [13–17]. Earlier we have shown that glutathionylation of Na,K-ATPase catalytic *α*-subunit is a determinant of the enzyme redox sensitivity [13]. Four cysteines of Na,K-ATPase *α*-subunit can undergo regulatory glutathionylation in case of GSSG increase, which leads to reversible inactivation of the enzyme, preventing exhaustion of ATP in the cells under oxidative stress. In addition to the regulatory glutathionylation of Na,K-ATPase *α*-subunit, its basal glutathionylation was found which is not removed by DTT [13]. This leads to the suggestion that the basally glutathionylated cysteine residues are located in a region of protein structure inaccessible to solvent. Basal glutathionylation was identified for the Na,K-ATPase *α*-subunit from various organisms, duck, mole rats, rabbit, and rat, in both cell lysates and purified enzyme preparations [13, 17, 18]. Basal glutathionylation is described for a variety of proteins: inhibitory kappa kinase beta IKK*β*, actin, and aldose reductase [5, 6]. Although basal glutathionylation of proteins is a widespread phenomenon, its role remains unclear. Despite the fact that the number of proteins with experimentally observed glutathionylation is growing rapidly [6], there is very small number of crystallographic structures with the identified bounded glutathione; that is, glutathione molecules are not reported in the existing X-ray structures of Na,K-ATPase *α*-subunit. Identification of glutathione in the structures of glutathionylated proteins is a common problem and a number of approaches are used to achieve this, as in the structure of mitochondrial ABC transporter Atm1 where authors compared the free and glutathione-bound protein structures to prove the presence of bonded glutathione [19]. They showed that there is unresolved electron density in the glutathione-bound protein, and it corresponds to the glutathione molecule. Based on this work, we have assumed that associated glutathiones in the X-ray structures of *α*-subunit of Na,K-ATPase should be shown as regions with unresolved density. We have analyzed all X-ray structures of the Na,K-ATPase *α*-subunit from pig kidney and found that there are a number of isolated cavities with unresolved electron density close to the relevant cysteine residues. Analysis of the structures showed that the unresolved density in the structure can be occupied by glutathione associated with cysteine residues. We have shown that acute hypoxia leads to increase of total glutathionylation level of Na,K-ATPase *α*-subunit; however, basal glutathionylation of *α*-subunit increases under prolonged hypoxia only. We have found that basal glutathionylation could be removed from fully denatured enzyme only. In this paper, we discuss the role of the basal glutathionylation and provide evidence supporting the view that this modification is cotranslational.

## 2. Materials and Methods

### 2.1. Modelling

**Figure 1:**
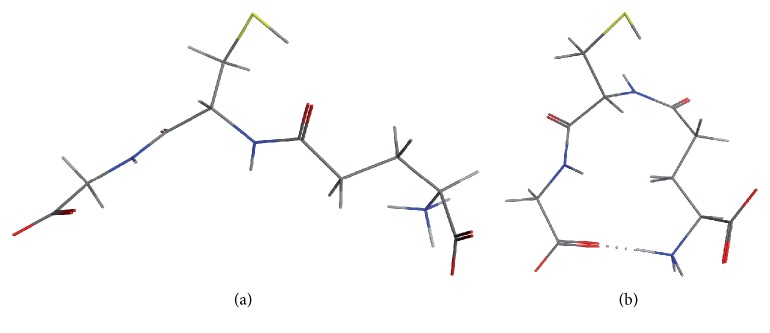
Three-dimensional models of glutathione used for the Na,K-ATPase, GSH modelling. (а) “Linear” model with elongated shape. (b) “Compact” model, structure forms a loop with hydrogen bond formed between the ends of the molecule.

Models of the glutathionylated Na,K-ATPase were constructed by COOT program using 3B8E, 3KDP, 3WGU, 3WGV, and 4HYT (PDB ids) structures as templates [20–22]. Two model structures of glutathione, the “linear” and “compact,” were built and minimized (Figure 1). In order to create models of the glutathionylated Na,K-ATPase, a corresponding model structure of glutathione was manually inserted into the cavity with unresolved electronic density (negative density and blob) using COOT. We assumed that the cavity was isolated when the distance between atoms of the residues forming the cavity was less than the sum of two van der Waals radii. Initially, GSH molecule has been placed in the cavity to verify conformity of the size of GSH molecule and the cavity. Subsequently, the GSH molecule was positioned in the cavity so as to form disulfide bond with a cysteine residue inside the cavity, and there were no structure overlaps. The resulting models of Na,K-ATPase with the S-S bonded glutathione molecule were locally minimized in the MMFF94x force field with the MOE program, version 2009.10. This force field accounts for atomic charges and hydrogen and ionic bonds between neighboring atoms. RMSD values of templates and glutathionylated models were calculated with MOE program (all atom types and parameters of symmetry were taken into account). Analysis of the presence of disulfide bonds in the Na,K-ATPase *α*-subunit structures was performed using DCCP program [23].

### 2.2. Cell Culture

Murine fibroblast cells (SC-1 cell line) were grown on DMEM media containing 10% FBS (Fetal Bovine Serum), 2 mM L-glutamine, 100 units/mL penicillin, and 100 mg/mL streptomycin at 37°C in a humid atmosphere with 5% CO_2_. SC-1 cells were grown either for 3.5 h or for 96 h, each at 20% and 0.2% pO_2_ (gas mixture contains 5% CO_2_ and nitrogen). Cells were lysed in cold RIPA buffer (containing 1% Nonidet P-40, 1% sodium deoxycholate, 0.1% SDS, 150 mM NaCl, 0.2 mM phenylmethylsulfonyl fluoride, protease inhibitor cocktail 6 mg/mL, and 25 mM TrisHCl (pH 7.6)) for 60 minutes at 4°C. Cell lysates were cleared by centrifugation at 16 000 g for 10 minutes. Supernatants were collected. Cell lysates were incubated with or without 25 mM reducing agent TCEP (Tris(2-carboxyethyl)-phosphine) (Thermo Scientific) soluble in Tris buffer, pH 7.4, during 30 min at 37°C. Proteins were separated on SDS-PAGE and then *α*1-subunit of Na,K-ATPase was detected by immunoblotting.

### 2.3. Immunoblotting

The level of S-glutathionylation of Na,K-ATPase *α*1-subunit was estimated using immunoblotting. Proteins of cell lysates were separated on SDS-PAGE and transferred to a PVDF membrane. After the blocking procedure, mouse monoclonal anti-glutathione antibody (Chemicon Millipore, MAB5310) was added. Mouse monoclonal anti-Na,K-ATPase *α*1 antibody clone C464-6 (Upstate Millipore) was applied to detect total amount of *α*1-subunit, followed by horseradish peroxidase-conjugated secondary antibodies. Membrane was stained using a commercial kit SuperSignal*™* West Femto Maximum Sensitivity Substrate (Thermo Scientific) and chemiluminescence was detected using Bio-Rad ChemiDoc MP instrument. Densitometric analysis was performed by Image Lab (Bio-Rad) program and the results were represented as ratio of glutathionylated *α*-subunit to total *α*-subunit band intensity ((GSS-*α*1)/total *α*1). The comparison was made between samples applied on the same membrane. The ratio of the bands (GSS-*α*1)/total *α*1 in control was taken as 1.

### 2.4. Immunoprecipitation

Immunoprecipitation was performed on lysates of SC-1 cells. Anti-Na,K-ATPase *α*1 antibody (3 *μ*g) was added to the cell lysate and the sample was incubated for 15 minutes at 4°С and constant agitation. The resulting immune complex was added to a tube containing protein A agarose and incubated for 2 h at 4°С and constant agitation. The sample was then centrifuged for 1 min at 15000 g and the supernatant was removed. The precipitate was washed with PBS for three times and then heated with 4x Laemmli buffer containing 8 M urea and 8% SDS at 80°С for 5 min to elute the protein. The sample was centrifuged and the supernatant was collected. The supernatant containing Na,K-ATPase *α*1-subunit was divided into two parts. One part was incubated without and another with 25 mM TCEP dissolved in Tris buffer, pH 7.4, during 30 min at 37°C. Glutathionylation of Na,K-ATPase *α*1-subunit in samples was detected by immunoblotting.

## 3. Results and Discussion

### 3.1. Structures of Na,K-ATPase from Pig Kidney Have Isolated Cavities with Unresolved Density near Several Cysteine Residues

There are eight structures of Na,K-ATPase from pig kidney, but only five of them have resolution better than 4 Å (Table 1), which allow for identifying the isolated cavities containing unresolved electron density near cysteine residues. In these structures, the isolated cavities were identified close to cysteine residues Cys 204, Cys 242, Cys 336, Cys 349, Cys 367, Cys 421, Cys 452, Cys 456, Cys 457, Cys 511, Cys 549, Cys 577, Cys 656, Cys 599, and Cys 698 of Na,K-ATPase *α*-subunit (Table 2). It has been found that the SH groups of pairs of cysteine residues were oriented into the same cavities: Сys 204, Cys 242; Cys 367, Cys 698; Cys 452, Cys 456 (457); Cys 511, Cys 549. However, using the DSSP program [23], we have shown that there are no disulfide bonds in all structures of Na,K-ATPase *α*-subunit.

To identify glutathione molecules bonded with cysteine residues, we searched the cavities with unresolved electron density. Unresolved density is a region of relatively high residual electron density that cannot be explained by presence of water [25]. Large areas of unresolved density were found in closed cavities near cysteines by COOT program [25]. The greatest number of areas with unresolved density, similar in shape and size to glutathione molecule, was found near the residues Cys 204, Cys 242; Cys 367, Cys 698; Cys 452, Cys 456, Cys 457; and Cys 599. It should be noted that there is no unresolved density near Cys 421 in all these structures, which coincides with the mass spectrometry data indicating that cysteine residue 421 (Cys 423 of duck *α*1) has never been found S-glutathionylated [13].

We have analyzed which of the cysteine residues in the pair may be more accessible for glutathionylation. According to [4, 26, 27], cysteine residues located close to positively charged residues (arginine, lysine, and histidine) are more accessible for glutathionylation because they attract the electron density, increasing the probability of glutathione binding. On the contrary, negatively charged residues close to cysteine reduce the probability of glutathionylation. Analysis of the amino acid composition near Cys residues showed that in the pairs of Cys residues pointing into the same cavity one of the cysteines had a positively charged amino acid in its close vicinity, whereas the other Cys had a negatively charged residue (Table 3). Considering these data, cysteine residues 204, 452, 599, and 698 are preferable for glutathionylation. The mass spectrometry data obtained earlier confirmed basal glutathionylation of residues 204 and 698 [13], but it was not possible to assess glutathionylation of residues 452 and 599.

### 3.2. Glutathione Fits into the Identified Isolated Cavities and Areas with Unresolved Electron Density

**Figure 2:**
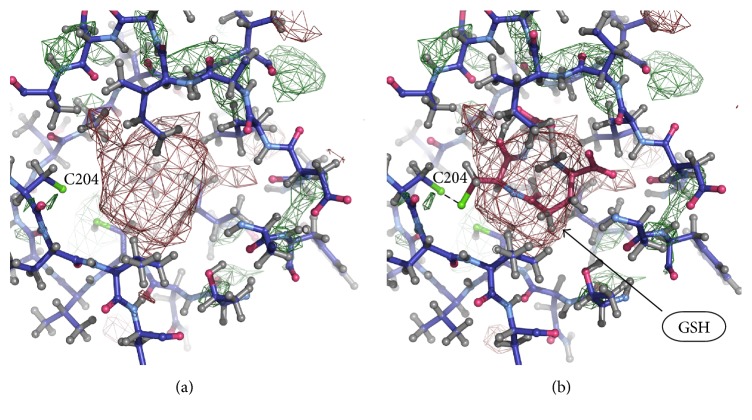
Part of the Na,K-ATPase *α*1-subunit near Cys204 and Cys242 (PDB code 3WGU). (a) Unresolved density in the isolated cavity. (b) GSH incorporated into the unresolved density.

**Figure 3:**
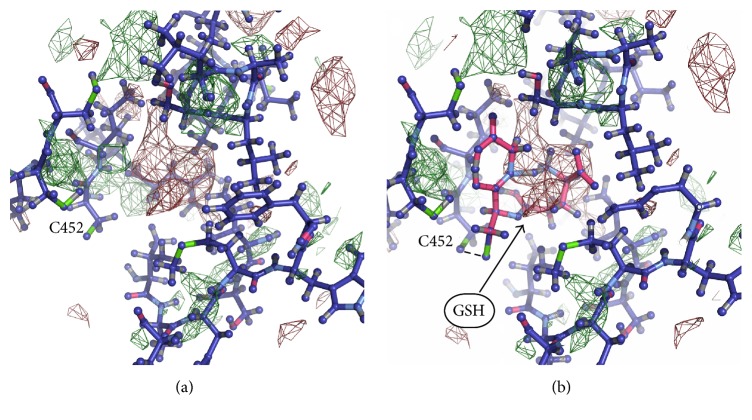
Part of Na,K-ATPase *α*1-subunit near Cys452 and Cys456 (PDB code 3WGU). (a) Unresolved density in the isolated cavity. (b) GSH incorporated into the unresolved density.

**Figure 4:**
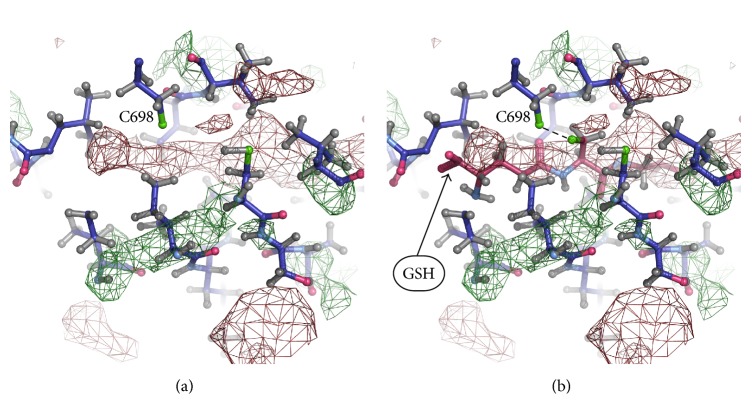
Part of Na,K-ATPase *α*1-subunit near Cys367 and Cys698 (PDB code 3WGU). (a) Unresolved density in the isolated cavity. (b) GSH incorporated into the unresolved density.

**Figure 5:**
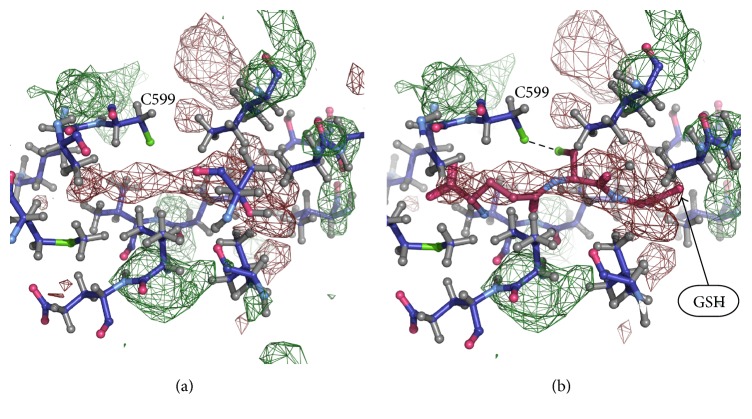
Part of Na,K-ATPase *α*1-subunit near Cys599 (PDB code 3WGU). (a) Unresolved density in the isolated cavity. (b) GSH incorporated into the unresolved density.

Models of the Na,K-ATPase *α*-subunit with glutathione in isolated cavities were built manually using COOT to verify fitting of glutathione molecules to the cavities. For this purpose, two models of glutathione molecule were used (Figure 1), which were placed in isolated cavities avoiding overlap with the resolved parts of structure. Glutathione molecule was placed in isolated cavities to fit unresolved densities, and thiol group of glutathione has been directed towards the thiol group of a cysteine in the cavity. Areas of unresolved electron density, large enough to insert a glutathione molecule, were found in a number of isolated cavities in Na,K-ATPase near the cysteine residues listed in Table 4. It was found that the electron density capable of accommodating glutathione molecule by shape and size is located in cavities near the cysteine residues Cys 204, Cys 242 (Figure 2) (3WGU); Cys 452, Cys 456, and Cys 457 (Figure 3) (3WGU, 3KDP, and 3B8E); Cys 698, Cys 367 (Figure 4) (3WGU); and Cys 599 (Figure 5) (3WGU, 3KDP, 3B8E, and 4HYT) (Table 4). The role of these Cys residues in the functioning of the enzyme was evaluated previously with point mutagenesis [24]. Replacing one of Cys 367 and Cys 698 residues or all three Cys residues (452, 456, and 457) with alanine or serine resulted in reduced activity by more than 75%, while replacing Cys 204 and Cys 599 residues resulted in reduced activity by more than 50% (Table 4). This indicates that these Cys residues are not critical for the enzyme activity but their glutathionylation could play an important role in regulating the protein function. Substitution of Cys 242 to Ala or Ser residues was lethal to the cells, suggesting the central role of this residue for the functioning of Na,K-ATPase. According to our data, Cys 242 is one of the residues undergoing regulatory glutathionylation in oxidative stress [13].

The mutagenesis data allowed for concluding that the presence of disulfide bridges is not required for folding of the Na,K-ATPase *α*-subunit and its subsequent activity [24]. Indeed, analysis of the available structures shows that there are no disulfide bonds between the cysteine residues. Moreover, according to our data, closely located cysteine residues which could form disulfide bond are directed into the cavity containing glutathione. Thus, it can be hypothesized that glutathionylation prevents formation of the intramolecular SS bridges.

### 3.3. Energy Minimization of the Models

To verify the obtained data, an SS bond has been formed between the glutathione molecule and the nearest cysteine residue and energy minimization of the obtained models carried out using the MOE program. After minimization, in 8 out of 9 models, glutathione not only stayed within the unresolved density but has moved to a more consistent location in the given density. Only in a single case out of nine, the glutathione molecule after minimization was partially located outside the unresolved density (3WGU, Cys 452). Changes in the Na,K-ATPase structure after minimization are given in Table 5. RMSD between the initial and minimized Na,K-ATPase structures is in the range of 0.05–0.09 Å, indicating that that minimization with the bound glutathione does not result in significant changes of the Na,K-ATPase structure.

We have compared the distances between sulfur atoms in the pairs of the cysteines in the initial structure of the protein (3WGU) and in the model structures (obtained by us) with glutathione attached to one of the cysteine residues (Table 6). This comparison revealed that glutathione binding has little effect on the distance between sulfur atoms in the Cys pairs. After local minimization, RMSD value between the initial and glutathionylated Na,K-ATPase structures was smaller then RMSD value between the initial structure of Na,K-ATPase and Na,K-ATPase with Cys-S-S-Cys bridges (Table 5). Thus, shifting cysteine residues towards each other until the distance between them reached SS bridge length (2.04 Å) and subsequent formation of disulfide bridges make greater changes in the structure of the molecule than incorporation of glutathione. Introduction of glutathione in the regions of protein with unresolved density has almost no effect on protein structure and location of cysteine residues determined by crystallography.

### 3.4. Prolonged Incubation of Cells under Hypoxic Conditions Alters the Basal Level of Na,K-ATPase Glutathionylation

**Figure 6:**
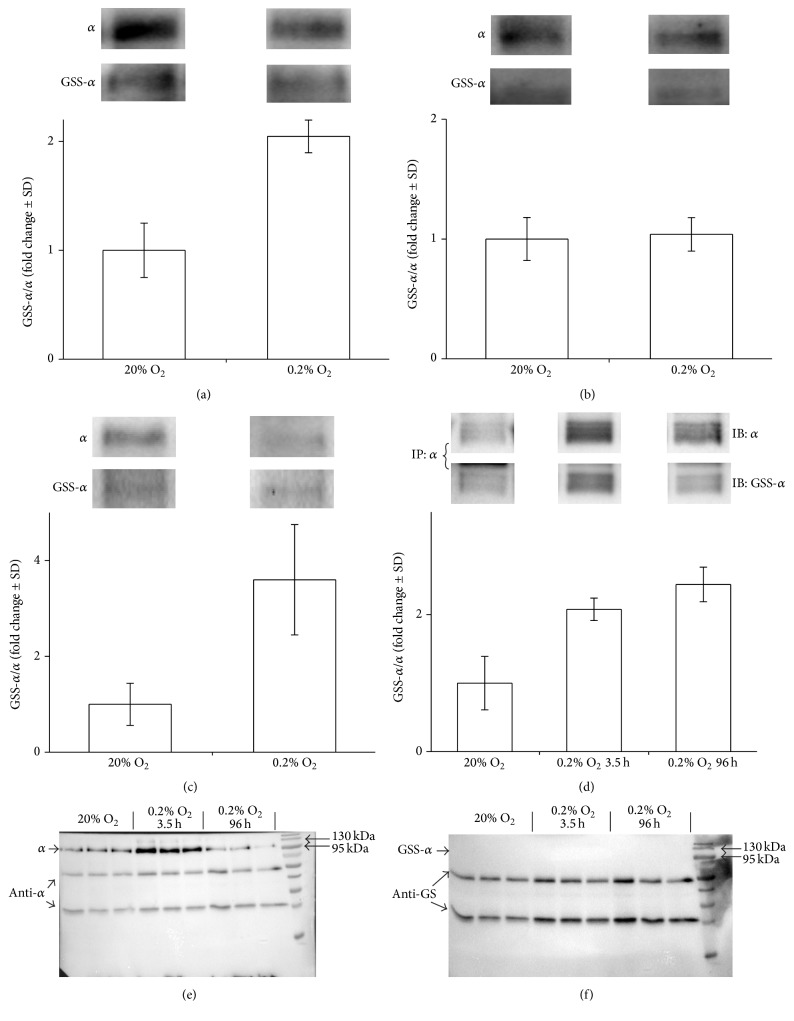
S-glutathionylation of *α*1-subunit of Na,K-ATPase after acute and prolonged hypoxia before and after TCEP (25 mM) treatment. SC-1 cells were grown either for 3.5 h ((a), (b)) or for 96 h (c), each at 20% and 0.2% pO_2_. Cell lysates were incubated with ((b), (c)) or without (a) 25 mM TCEP during 30 min at 37°C and *α*1-subunit of Na,K-ATPase was detected by immunoblotting (IB). (d) *α*1-Subunit of Na,K-ATPase was immunoprecipitated (IP) from cell lysates by anti-*α*1 antibodies and glutathionylation was detected with anti-glutathione (anti-GS) antibodies. The original immunoblotting readouts are presented above. Bars represent changes in the S-glutathionylated (GSS-*α*/*α*) form of the protein normalized to its total amount. *n* = 3, mean ± SD. ((e), (f)) Immunoblots of Na,K-ATPase *α*1-subunit after immunoprecipitation with anti-*α*1 antibodies and TCEP (25 mM) treatment in denaturing conditions (8 M urea, 8% SDS). (e) Detection of total *α*1-subunit using anti-*α*1 antibodies. (f) Detection of glutathionylated *α*1-subunit using anti-GS antibodies.

It can be hypothesized that the basal glutathionylation depends on the redox status of cells during protein folding. To test this, we incubated SC-1 cells at 0.2% and 20% pO_2_ within 3.5 h and 96 h. Then, cell lysates were treated with the reducing agent TCEP (25 mM) for 30 min. Acute hypoxia (3.5 h) leads to increase of total glutathionylation level of Na,K-ATPase *α*-subunit (Figure 6(a)) that corresponds to our data, obtained earlier [13, 28]. However, basal glutathionylation of *α*-subunit does not change at these conditions (Figure 6(b)). In contrast, at prolonged hypoxia (96 h), basal level of Na,K-ATPase *α*-subunit glutathionylation was significantly higher than that under 20% pO_2_ (Figure 6(c)). Since the basal glutathionylation is not removed by reducing agents and the glutathionylated cysteines are located in isolated cavities, we assume that the reaction of glutathionylation occurs during protein folding. If so, then the basal glutathionylation can be removed only from the unfolded protein. We performed immunoprecipitation of Na,K-ATPase *α*-subunit from lysates of SC-1 cells incubated at 0.2% and 20% pO_2_ within 3.5 h and 96 h. Analysis of immunoprecipitated *α*-subunit revealed increasing of its glutathionylation under hypoxic conditions (Figure 6(d)). Then, we treated Na,K-ATPase *α*-subunit obtained by immunoprecipitation with TCEP under denaturing conditions (8 M urea and 8% SDS). In this case, the glutathionylation was fully removed (Figures 6(e) and 6(f)). Based on these data, we can conclude that the basal glutathionylation is a cotranslational modification which, for example, is necessary to prevent the formation of disulphide bridges between the neighboring cysteine residues during protein folding. Formation of disulfide bridges can increase rigidity of the structure and prevent conformational lability of the molecule. In particular, residues Cys 204 and Cys 242 are located in the actuator domain of Na,K-ATPase that performs large amplitude transitions during the catalytic cycle, which would be impossible if SS bridges were formed between these residues. In addition, the formation of SS bridge between Cys 204 and Cys 242 residues will prevent the exposition of SH group of Cys 242 to the solvent from the cavity and its regulatory glutathionylation. It is also possible that glutathionylation is necessary for correct protein folding. Only glutathione and oxidized glutathione are required for glutathionylation of *α*-subunit of Na,K-ATPase [13] without any additional enzymes and cofactors. Thus, the ability of SH group to undergo glutathionylation directly depends on the redox state of cells [5]. Reduced SH groups interact with GSSG, the level of which increases in oxidative stress. In the case of oxidation of thiol groups to SOН, they interact with GSH, the concentration of which in cytosol is 1–5 mM.

**Figure 7:**
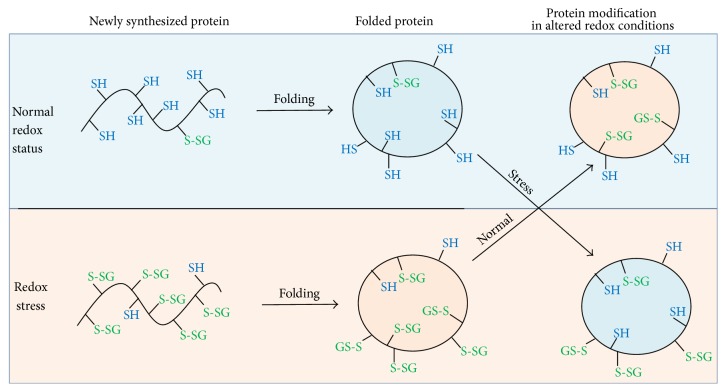
Schematic representation of Na,K-ATPase glutathionylation depending on intracellular redox status. At normal redox status, the level of GSH is about 100 times higher than GSSG. Under these conditions, during biosynthesis, the proteins are slightly glutathionylated. Redox stress leads to the shift in GSH/GSSG ratio that induces protein glutathionylation. At normal redox status and at redox stress, the basal levels of glutathionylation (glutathionylation of solvent-inaccessible cysteine residues) are different. Subsequent change in the redox status does not affect basal glutathionylation, which demonstrates that the protein “memorizes” a cellular redox state during its biosynthesis. In contrast, glutathionylation of the solvent-accessible cysteine residues (regulatory glutathionylation) depends on the current redox status of cell.

Unlike the genetic code and histone code that underlie information storage and utilization, the epigenetic code and redox code modulate operation of the genetic and histone codes in the organizational structure, differentiation, and adaptation of an organism to the environment [29]. Redox signaling and redox control in multicellular organisms evolved and diversified with the increase in atmospheric O_2_ about 600 million years ago. We assume that cotranslational glutathionylation allows for “remembering” a cellular redox state during protein biosynthesis, and as a result proteins synthesized in different redox conditions will have different properties (Figure 7). This phenomenon, which can be termed “redox memory,” may be a necessary step of redox regulation and cell adaptation to different oxygen levels and other factors that change the intracellular redox status.

## 4. Conclusions

Our analysis of the available Na,K-ATPase structures has revealed isolated cavities with unresolved density near several cysteine residues in the catalytic *α*-subunit of the enzyme; these cavities correspond to the glutathione molecule. Identification of such unresolved density near cysteine residues can be prognosticated for other proteins that can undergo glutathionylation. Basal glutathionylation will depend on the redox status of cell at the point of protein synthesis (Figure 7). Consequently, depending on the redox status of cell, proteins with diverse levels of glutathionylation will be synthesized, and such proteins accordingly will have different properties. Since basally glutathionylated residues are not accessible to the deglutathionylating agents, the pattern of basal glutathionylation reflects the redox status of the cell at the point of protein folding.

## Figures and Tables

**Table 1:** Structures of Na,K-ATPase from pig kidney with resolution better than 4 Å.

PDB ID	3B8E	3KDP	3WGU	3WGV	4HYT

Resolution	3.50 Å	3.50 Å	2.80 Å	2.80 Å	3.40 Å

Species, organ	Pig kidney	Pig kidney	Pig kidney	Pig kidney	Pig kidney

Conformation	E2P	—	E1P	E1P with oligomycin	E2P with ouabain

Method	Vapor diffusion and hanging drop	Vapor diffusion	Vapor diffusion and hanging drop	Vapor diffusion and hanging drop	Hanging drop

pH	7.0	7.0	6.2	6.2	6.2

Temperature	292.0 K	292.0 K	283.0 K	283.0 K	292.0 K

Details	14% PEG 2000 mme, 0.2 M choline chloride, 4% glycerol, 4% MPD, 0.04 M DTT, and 0.1–0.4% beta-DDM	14% PEG 2000 mme, 0.2 M choline chloride, 4% glycerol, 4% MPD, 0.04 M DTT, and 0.1–0.4% beta-DDM	Na,K-ATPase was incubated with a buffer, 150 mM NaCl, 1 mM AlCl_3_, 4 mM NaF, 4 mM ADP, 3 mM MgCl_2_, 2 mM glutathione, and 20 mM MOPS/n-methyl-D-glucamine (NMDG), pH 7.1, and treated with 1.95% (w/v) octaethylene glycol mono-n-dodecyl ether (C12E8) at a mass ratio (C12E8/protein) of 1.3 and separated from the insoluble fraction by centrifugation at 200.000 g and 10 uC; 17.5% PEG 2000 mme, 10% glycerol, 200 mM NaCl, 50 mM MES-NMDG	Na,K-ATPase was incubated with a buffer, 150 mM NaCl, 1 mM AlCl_3_, 4 mM NaF, 4 mM ADP, 3 mM MgCl_2_, 2 mM glutathione, and 20 mM MOPS/n-methyl-D-glucamine (NMDG), pH 7.1, with 0.25 mM oligomycin A and treated with 1.95% (w/v) octaethylene glycol mono-n-dodecyl ether (C12E8) at a mass ratio (C12E8/protein) of 1.3 and separated from the insoluble fraction by centrifugation at 200.000 g and 10 uC; 17.5% PEG 2000 mme, 10% glycerol, 200 mM NaCl, 50 mM MES-NMDG	16-17% PEG 2000 mme, 10% glycerol, 200 mM MgCl_2_, 100 mM MES-NMDG, pH 6.2, 100 mM urea, 5% *tert*-butanol, 5 mM DTT, and 1.6 mM sucrose monodecanoate

Reference	[20]	[20]	[21]	[21]	[22]

**Table 2:** Unresolved electron density close to cytosolic cysteine residues in X-ray structures of Na,K-ATPase *α*-subunit.

PDB ID/Cys position	3WGU	3WGV	3KDP	4HYT	3B8E	S-glutathionylation^a^
204, 242	Density^b^	Density	Small size of density^c^	Small size of density	Small size of density	+
336	Distant density^d^	Distant density	Density	Density	Distant density	+
349	Fragmented density^e^	Fragmented density	nd^f^	nd	nd	+
367, 698	Density	Fragmented density	Distant density	nd	nd	+
421	nd	nd	Small size of density	nd	nd	−
452, 456	Density	Fragmented density	Density	Fragmented density	Density	+
457	Surface	Surface	Surface	Surface	Surface	+
511, 549	Density	Small size of density	nd	nd	nd	+
656	Fragmented density	Fragmented density	nd	nd	nd	+
599	Density	Fragmented density	Density	Density	Density	Not detected
577	Surface	Surface	Surface	Surface	Surface	Not detected

^a^According to MALDI-TOF mass spectrometry data in [13].

^b^“Density”: unresolved density that can fit by glutathione.

^c^“Small size of density”: unresolved density but too small to fit glutathione.

^d^“Distant density”: unresolved electron density at a distance of more than 5 Å from Cys (glutathione fits density, but the density is far from the residue).

^e^“Fragmented”: no intact unresolved density.

^f^“nd”: no unresolved density close to the residue.

**Table 3:** Amino acid composition of porcine Na,K-ATPase *α*-subunit near Cys residues.

Cys position	Neighboring amino acid residues
204	NGCKV
242	TNCVE
367	TICSD
698	EGCQR
452	LKCIE
456, 457	ELCCGS
599	GKCRS

**Table 4:** Cysteine residues with unresolved GSH-like electron density and effect of their substitution by alanine or serine on Na,K-ATPase activity.

PDB ID/Cys position	3WGU	3WGV	3KDP	4HYT	3B8E	Na,K-ATPase activity according to [24], mutant/wild type, %
204	Density	Density	Small density	Small density	Small density	49.2
242						—^a^

367	Density	Fragmented density	Distant density	nd	nd	23.4
698						22.9

452	Density	Fragmented density	Density	Fragmented density	Density	25.3^b^
456						

599	Density	Fragmented density	Density	Fragmented Density	Density	43.5

Abbreviations and all other details are given in the legend of Table 2.

^a^Cells with Cys 242 Ala or Cys 242 Ser mutant of Na,K-ATPase did not survive under ouabain selective pressure.

^b^Activity of Cys 452, Cys 456, and Cys 457 Ser mutant of Na,K-ATPase.

**Table 5:** RMSD between initial (PDB ID 3WGU) and model structures of Na,K-ATPase with SS bridges between pair of Cys residues (SS RMSD) and between Cys residue and glutathione (Cys-SSG RMSD) after local and global minimization.

Pairs of Cys residues	SS RMSD, Å local minimization^a^	Glutathionylated Cys	Cys-SSG RMSD, Å local minimization^a^
204–242	0.12	204	0.09

367–698	0.14	698	0.08

452–456	0.12	452	0.05

^a^Local minimization: minimization within a radius of 4.5 Å from the thiol groups of each cysteine residue in MMFF94x force field.

**Table 6:** Distance between Cys thiol groups in initial Na,K-ATPase structure (3WGU) and in model structures with glutathionylated Cys residues.

Pairs of Cys residues	Distance between Cys thiol groups in initial structure, Å	Glutathionylated Cys	Distance between Cys thiol groups in glutathione bound structure, Å
204–242	3.82	204	3.56
		242	3.46

367–698	4.10	367	5.47
		698	5.14

452–456	4.22	452	3.70
		456	5.63
